# Upsurge in the construction of chiral nanomaterials

**DOI:** 10.1039/d1sc90259h

**Published:** 2022-01-18

**Authors:** Zhiyong Tang, Laura Na Liu

**Affiliations:** CAS Key Laboratory of Nanosystem and Hierarchical Fabrication, CAS Center for Excellence in Nanoscience, National Center for Nanoscience and Technology Beijing 100190 People’s Republic of China zytang@nanoctr.cn; 2nd Physics Institute, University of Stuttgart 70569 Stuttgart Germany na.liu@pi2.uni-stuttgart.de

## Abstract

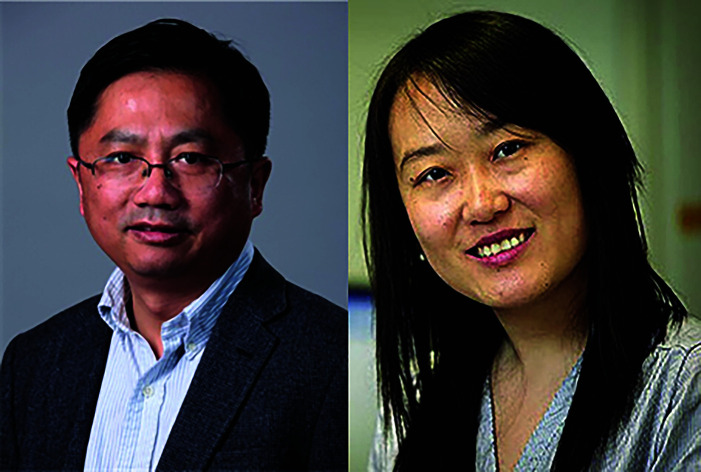

Owing to its fast development, chiral nanoscience has become one of the core research topics in chemistry, physics, medicine and materials science. Recently, many efforts have been focused on constructing new types of chiral nanomaterials with unique optical activity. This themed issue highlights the state-of-the art progress in this field, based on a series of invited and selected articles published in *Chemical Science*. These articles cover diverse nanomaterials, including organic, inorganic, organic–inorganic hybrid and superstructured nanomaterials. The details based on the composition are as follows:

On the topic of organic nanomaterials, one can refer to Tomoki Ogoshi *et al.*’s work on constructing pillar[5]arene-based chiral nanotubes *via* pre-regulation of the building blocks’ chirality (DOI: 10.1039/D1SC00074H), the surface-induced enantiomorphic crystallization of achiral fullerene derivatives in thin films done by Keisuke Tajima *et al.* (DOI: 10.1039/D0SC01163K), and chiral polymer hosts for circularly polarized electroluminescence devices realized by Changsoon Kim, Youngmin You and coworkers (DOI: 10.1039/D1SC02095A).

Specific to organic nanomaterials with chiral aggregate-induced emission (AIE) properties, Yanhua Cheng, Ben Zhong Tang and coworkers reported the polymorph selectivity of an AIE luminogen under nano-confinement to visualize polymer microstructures (DOI: 10.1039/C9SC04239C). Minghua Liu, Shimei Jiang and coworkers presented multicolor tunable circularly polarized luminescence in a single AIE system (DOI: 10.1039/C9SC05643B). Qinghua Lu, Hailiang Zhang, Quan Li and coworkers showed solvent polarity driven helicity inversion and circularly polarized luminescence in chiral AIE fluorophores (DOI: 10.1039/D0SC04179C). A corresponding review about the chiral assembly of organic luminogens with AIE properties was given by Hai-Tao Feng, Ben Zhong Tang and coworkers (DOI: 10.1039/D1SC02305E).

On the topic of inorganic nanomaterials, Masahiro Ehara, Takuya Nakashima and coworkers reported enantioseparation and chiral induction in Ag_29_ nanoclusters with intrinsic chirality (DOI: 10.1039/C9SC05299B). Yoshitaka Aramaki, Takashi Ooi, Masakazu Nambo, Cathleen M. Crudden and coworkers introduced the synthesis and enantioseparation of chiral Au_13_ nanoclusters protected by bis-N-heterocyclic carbene ligands (DOI: 10.1039/D1SC03076K). Georg H. Mehl showed the development of two helices from one chiral center in self-organized disc-shaped chiral nanoparticles (DOI: 10.1039/D0SC05100D). A review on template-assisted self-assembly of achiral plasmonic nanoparticles into chiral structures was written by Luis M. Liz-Marzán (DOI: 10.1039/D1SC03327A).

On the topic of organic–inorganic hybrid nanomaterials, Shu Kobayashi prepared heterogeneous Rh and Rh/Ag bimetallic nanoparticle catalysts immobilized on chiral polymers with high-to-excellent yields and enantioselectivities (DOI: 10.1039/C9SC02670C). Xiaogang Qu fabricated a series of stereoselective nanozymes (Fe_3_O_4_@poly(AA)) using a ferromagnetic nanoparticle yolk as the catalytic core and amino acid-appended chiral polymer shell as the chiral selector (DOI: 10.1039/D0SC03082A). Yongsheng Zhao, Chuanlang Zhan, Jiannian Yao and coworkers reported lanthanide MOFs for inducing the molecular chirality of achiral stilbazolium with strong circularly polarized luminescence and efficient energy transfer for color tuning (DOI: 10.1039/D0SC02856H).

On the topic of superstructures, Zeyuan Dong constructed a helical supramolecular polymer nanotube by manipulating strong noncovalent interactions (DOI: 10.1039/C9SC02336D). De-Liang Long, Leroy Cronin and coworkers fabricated peptide sequence mediated molybdenum blue nanowheel superstructures (DOI: 10.1039/D0SC06098D). Kazuhiko Nakatani, Chikara Dohno, Ben L. Feringa and coworkers designed a photoswitchable DNA glue with high regulatory function and supramolecular chirality transfer (DOI: 10.1039/D1SC02194J). A related review of hierarchical self-assembly into chiral nanostructures was presented by Minghua Liu (DOI: 10.1039/D1SC03561D).

The above articles and reviews provide a complete picture of the construction of various chiral nanomaterials. We hope that the readers will quickly grasp the entire concept of this field with the help of this themed issue.

## Supplementary Material

